# Ultrasound-Triggered Enzymatic Gelation

**DOI:** 10.1002/adma.201905914

**Published:** 2020-01-10

**Authors:** Valeria Nele, Carolyn E. Schutt, Jonathan P. Wojciechowski, Worrapong Kit-Anan, James J. Doutch, James P. K. Armstrong, Molly M. Stevens

**Affiliations:** Department of Materials, Department of Bioengineering and Institute of Biomedical Engineering Imperial College London, Prince Consort Road, London SW7 2AZ, UK; ISIS Neutron and Muon Source, STFC, Rutherford Appleton Laboratory, Didcot OX11 ODE, UK; Department of Materials, Department of Bioengineering and Institute of Biomedical Engineering, Imperial College London, Prince Consort Road, London SW7 2AZ, UK

**Keywords:** enzymes, hydrogels, liposomes, microbubbles, ultrasound

## Abstract

Hydrogels are formed using various triggers, including light irradiation, pH adjustment, heating, cooling, or chemical addition. Here, a new method for forming hydrogels is introduced: ultrasound-triggered enzymatic gelation. Specifically, ultrasound is used as a stimulus to liberate liposomal calcium ions, which then trigger the enzymatic activity of transglutaminase. The activated enzyme catalyzes the formation of fibrinogen hydrogels through covalent intermolecular crosslinking. The catalysis and gelation processes are monitored in real time and both the enzyme kinetics and final hydrogel properties are controlled by varying the initial ultrasound exposure time. This technology is extended to microbubble–liposome conjugates, which exhibit a stronger response to the applied acoustic field and are also used for ultrasound-triggered enzymatic hydrogelation. To the best of the knowledge, these results are the first instance in which ultrasound is used as a trigger for either enzyme catalysis or enzymatic hydrogelation. This approach is highly versatile and can be readily applied to different ion-dependent enzymes or gelation systems. Moreover, this work paves the way for the use of ultrasound as a remote trigger for in vivo hydrogelation.

Hydrogels are hydrated, 3D polymeric networks that are widely used for applications in tissue engineering, drug delivery, soft robotics, and bioelectronics.^[1–6]^ The base materials encompass a broad range of hydrophilic homopolymers, copolymers, or macromers, which can be natural (e.g., collagen, alginate, fibrin), fully synthetic (e.g., poly(ethylene glycol), poly(vinyl alcohol), poly(acrylic acid)), or semisynthetic (e.g., methacrylate-, tetrazine-, norbornene-modified biopolymers).^[7]^ Hydrogels are formed through sol–gel transitions mediated by the formation of various noncovalent or covalent bonds. For instance, many hydrogels are crosslinked by ions, small molecules, or peptides, which form chemical bonds that bridge adjacent polymer chains.^[8,9]^ However, the need for a second component to be added to the system presents challenges for many applications, in particular in vivo gelation. Hydrogelation can also be initiated by changing environmental conditions, such as temperature^[10]^ or pH.^[11]^ These stimuli can be used to directly alter the chemical environment of the material through changes in noncovalent interactions, or alternatively, can be used to trigger the release of chemical factors that initiate gelation. This strategy is used for injectable formulations that are designed to gel under physiological conditions, however, these systems are typically limited by poor spatiotemporal control. One method that can achieve high spatiotemporal precision is the use of UV or blue light irradiation to photocrosslink synthetic or semisynthetic hydrogels.^[12]^ Yet, photocrosslinking applications can be hindered by the common need for radical photoinitiators, as well as the limited tissue penetration of light at these wavelengths.^[13,14]^

One potentially valuable trigger for hydrogelation is ultrasound: mechanical pressure waves that oscillate at high frequency (≥20 kHz) and produce a range of thermal and non-thermal effects. For example, the absorption of ultrasonic energy by the surrounding medium can produce localized hyperthermia and acoustic streaming,^[15]^ while ultrasound pressure oscillations can generate acoustic radiation forces^[16]^ and modulate the nucleation, growth, and oscillation of gaseous microbubbles.^[17,18]^ These effects have been exploited for a variety of biomedical applications: to pattern cell arrays for in vitro tissue engineering,^[19]^ to stimulate osteogenesis for accelerated bone fracture healing,^[20]^ to temporarily disrupt the blood–brain barrier for systemic drug delivery,^[21]^ to induce localized hyperthermia for ablation therapy,^[22]^ and to visualize anatomical structure and blood perfusion using ultrasonography.^[23,24]^ Ultrasound has also been widely used as an in vitro and in vivo trigger for releasing drugs from liposomes,^[25,26]^ nanoemulsions,^[27]^ and microbubbles.^[28]^ These studies demonstrate that ultrasound offers high biocompatibility, excellent tissue penetration, and the capacity for spatiotemporal and remote controlled payload release.^[29]^

Given these clear benefits, it is surprising that ultrasound has been largely overlooked as a trigger for hydrogelation. In 2008, Park and Kim used ultrasound to generate radicals that could initiate the formation of 2’-deoxyadenosine-based hydrogels. However, the use of radical species to mediate gelation can pose cytotoxicity issues that can restrict biomedical applications.^[31]^ In this article, we demonstrate that hydrogelation can be initiated using ultrasound-triggered enzyme catalysis. Specifically, we used ultrasound to release calcium ions from liposomes in order to trigger the catalysis of transglutaminase (Figure 1a). The ultrasound-activated transglutaminase can then catalyze intermolecular covalent crosslinking between the lysine and glutamine sidechain residues of soluble fibrinogen molecules, in order to produce fibrinogen hydrogels (Figure 1b). We were able to leverage a high degree of control over these processes, with the calcium ion release, catalysis rate, and hydrogelation rate all shown to be dependent upon the ultrasound exposure time. We further extended the capabilities of this technology by conjugating calcium-loaded liposomes to the surface of gaseous microbubbles that have been widely studied as an aid for in vivo drug delivery.^[32–34]^ These microbubble–liposome conjugates displayed an enhanced response to the applied acoustic field and could also be used for ultrasound-triggered hydrogelation. Overall, these methods enable on-demand, ultrasound-triggered enzyme catalysis and enzymatic hydrogelation without the use of radical species or stimuli-responsive polymers. Indeed, the underlying principles are readily applicable to a range of ion-dependent enzymes and hydrogel systems. This versatility presents a host of opportunities for in vitro and in vivo applications in materials science, biomedical engineering, drug delivery, and beyond.

Our field-responsive system required a stable formulation of calcium-loaded liposomes that could release their payload upon ultrasound exposure. We selected a liposome formulation consisting of two lipids: 1,2-dipalmitoyl-*sn*-glycero-3-phosphocholine (DPPC) doped with 1 mol% 1,2-distearoyl-*sn*-glycero-3-phosphoethanolamine-*N*-[biotinyl(polyethylene glycol)-2000] (DSPE-PEG_2000_ biotin). DPPC membranes are in a gel phase at temperatures lower than 41°C and thus should provide high cargo retention prior to the ultrasound-triggered release of calcium ions. Meanwhile, the small fraction of biotinylated lipid served as a reactive handle for liposome functionalization. We selected an interdigitation-fusion vesicle method in order to produce liposomes with high intraluminal calcium loading.^[35]^ We hydrated the lipid mixture with aqueous CaCl_2_ to produce a polydisperse mixture of calcium-loaded multilamellar liposomes. We used ethanol to induce bilayer interdigitation and generate large unilamellar liposomes, which we then extruded to form small unilamellar liposomes. We analyzed the unextruded and extruded liposomes using small-angle neutron scattering and a lamellar model fit, which estimated bilayer thicknesses of 49.1 ± 0.1 and 50.9 ± 0.1 Å, respectively (Figure S1, Supporting Information). Meanwhile, we used cryogenic transmission electron microscopy to confirm that the liposomes were unilamellar before and after extrusion (Figure S2, Supporting Information). We further characterized the extruded liposome population using dynamic light scattering, which gave a hydrodynamic diameter of 122 ± 43 nm (Figure S3a, Supporting Information). This value correlated well with the liposome diameter of 144 ± 51 nm measured using nanoparticle tracking analysis (NTA; Figure S3b, Supporting Information).

We tested a range of CaCl_2_ concentrations during lipid hydration (0.2, 0.4, 0.6 m) and measured the liposomal calcium loading using an *ortho*-cresolphthalein complexone (*o*-CPC) colorimetric assay and NTA particle counting. We observed a 37% increase in the encapsulated calcium per liposome when the concentration of the CaCl_2_ solution was raised from 0.2 m ((3.3 ± 0.4) × 10^–19^ mol liposome^–1^) to 0.4 m ((4.6 ± 0.1) × 10^–19^ mol liposome^–1^). However, we observed a reduced yield of liposomes and a lower calcium loading at the highest tested concentration of 0.6 m CaCl_2_ ((0.5 ± 0.1) × 10^–19^ mol liposome^–1^) (Figure S4, Supporting Information). Based on these studies, we selected 0.4 m CaCl_2_ as the hydrating solution for all subsequent studies. We observed that our liposomes were stable against aggregation and uncontrolled calcium leakage, with no changes in hydrodynamic diameter and less than 2% of the encapsulated cargo released after 5 days at 25°C (Figure S5, Supporting Information). Having established this baseline, we then sought to assess whether we could trigger calcium ion release from the liposomes using ultrasound. This was based on the principle of acoustic cavitation, whereby ultrasound fields stimulate the formation and collapse of vapor bubbles, which produces shockwaves that can compromise liposomal membranes.^[36–38]^ For this study, we applied 20 kHz ultrasound at 20% amplitude and 25% duty cycle, with the exposure time varied between 1 and 50 s. Using these parameters, we were able to liberate up to 92% of the total encapsulated calcium, with a release quantity that was dependent on the ultrasound exposure time (Figure 2a). Importantly, there was no significant difference in the quantity of released calcium after incubating the liposomes for 5 days (Figure S6, Supporting Information).

The ability to controllably trigger calcium ion release using ultrasound opens up a wide range of possible applications. Here, we sought to apply this technology to modulate the catalytic activity of transglutaminase, a calcium-dependent enzyme. The transglutaminases are a class of enzymes that naturally catalyze isopeptide bond formation between the *ε*-amine of lysine and the sidechain amide of glutamine. Calcium ions play a key role by binding to transglutaminase and causing a conformational change in the enzyme structure, which exposes an active-site cysteine that can then initiate isopeptide bond formation.^[39,40]^ In order to measure this process, we monitored the fluorescence changes that occurred during the transglutaminase-catalyzed crosslinking between a model protein (*N,N*-dimethylcasein) and a fluorescent probe (dansylcadaverine). Specifically, we tested whether ultrasound-triggered calcium ion release could modulate transglutaminase activity over a 21 h period. We observed a dose-dependent enzyme activation when the ultrasound exposure time was varied between 1 and 10 s, and importantly, negligible catalysis without any ultrasound application (Figure 2b,c). We fitted the reaction kinetics to an asymptotic regression model *y* = *a* – b*^cx^, where *a* = 9.18, *b* = 9.07, *c* = 0.91, and *R*^2^= 0.95. The initial reaction rate increased linearly with increasing ultrasound exposure time and started to plateau at 10 s ultrasound exposure, which corresponded to 37% of liberated calcium.

Having established a method for ultrasound-triggered enzyme activity, we next investigated whether we could use ultrasound to initiate a hydrogelation process. Specifically, we hypothesized that the calcium ions released by ultrasound-exposed liposomes could be used to trigger the transglutaminase-catalyzed hydrogelation of fibrinogen. Transglutaminase catalyzes intramolecular and intermolecular fibrinogen crosslinking, with the latter used to form fibrinogen hydrogels.^[41]^ We applied ultrasound for 3, 10, or 50 s (20 kHz frequency, 25% duty cycle, 20% amplitude) to a liquid solution of calcium-loaded liposomes, and monitored the transglutaminase-catalyzed hydrogelation of fibrinogen using time-resolved rheology (1% strain, 1 rad s^–1^ frequency). We observed a relatively rapid gelation in all cases, with the elastic modulus (*G′*) exceeding the viscous modulus (*G′′*) in less than 1 min, including the time taken for loading the liquid onto the rheometer (Figure 2d–f). Indeed, due to the fast kinetics of the process, it was not possible to precisely capture the crossover point, at which *G′* > *G′′*. Faster or slower hydrogelation could be achieved simply by increasing or decreasing the transglutaminase concentration, respectively (Figure S7, Supporting Information). Collectively, these results indicate that hydrogelation kinetics can be tuned by changing either the enzyme concentration or the ultrasound exposure time. Importantly, the unexposed controls were liquid at 6 h, validating the role of ultrasound in the hydrogelation process (Figure S8, Supporting Information). The plateau elastic modulus (*G′*_p_) at the 5 h endpoint of the kinetic study was found to be dependent upon the initial ultrasound exposure time: 34, 55, and 177 Pa for 3, 10, and 50 s, respectively. These weak mechanical properties are typical of fibrinogen hydrogels.^[42]^ The elastic modulus could also be tuned by changing the fibrinogen concentration or by increasing the crosslinking time, e.g., an ultrasound-triggered gelation of a 33.6 mg mL^–1^ fibrinogen solution reached a *G′* > 1 kPa after 23 h of crosslinking (Figures S9 and S10, Supporting Information).

It should be noted that due to the fast catalysis and gelation rates, transglutaminase was added after exposure and immediately prior to fluorescence monitoring, which enabled us to resolve the early-stage changes required for kinetic analysis (see the Experimental Section). Since previous studies have shown that ultrasound can modulate enzyme activity,^[43,44]^ we sought to test the catalytic activity of transglutaminase after exposure to ultrasound (3, 10, 50 s), with dansylcadaverine and dimethylcasein added at different post-exposure time points (0, 24, 48, 72 h) (Figure S11, Supporting Information). These results showed that a 50 s exposure significantly and immediately decreased catalytic activity, while the 10 s exposure also reduced activity, but only after 24 h. The 3 s exposure had no significant effect on the enzyme activity at any of the time points tested. Given these results, we performed endpoint fluorescence and rheology measurements, which validated that ultrasound could still trigger enzyme activity and hydrogelation when all components were present during ultrasound exposure (Figures S12 and S13, Supporting Information).

Having successfully demonstrated ultrasound-triggered enzyme catalysis and hydrogelation using calcium-loaded liposomes, we sought to extend our capabilities by integrating our technology with ultrasound-responsive gaseous microbubbles, which have been used in drug delivery,^[45,46]^ ultrasound imaging,^[47,48]^ and thermal ablation.^[49,50]^ Conjugation of liposomes to microbubbles has previously been used to enhance the ultrasound-triggered release of liposomal cargo.^[51]^ Therefore, we investigated whether we could engineer microbubble–liposome conjugates capable of ultrasound-triggered hydrogelation of fibrinogen. We produced biotinylated microbubbles by hydrating a lipid film comprising 1,2-distearoyl-*sn*-glycero-3-phosphocholine, DSPE-PEG_2000_, and DSPE-PEG_2000_ biotin in a molar ratio of 86:9:5, and then pumping the solution with a mixture of perfluorohexane and air (see the Experimental Section). We used bright field microscopy to visualize the microbubbles (Figure S14, Supporting Information) and image analysis to measure a mean microbubble diameter of 2.5 ± 1.6 μm (Figure 3b). We conjugated liposomes to the surface of the microbubbles by using neutravidin to bind with the biotin moieties present on both components (Figure 3a). Using confocal fluorescence microscopy, we observed co-localization of fluorescently labeled liposomes on the surface of fluorescently labeled microbubbles, which indicated a successful conjugation (Figure 3c). Further insight was provided by structured illumination microscopy, a super-resolution imaging technique that revealed liposomes uniformly distributed across the microbubble surface (Figure 3d, Video S1, Supporting Information).

Using an *o*-CPC assay, we measured a calcium loading of (4.6 ± 0.6) × 10^–16^ mol per microbubble–liposome conjugate (Figure S15, Supporting Information), a sufficient quantity to test calcium release and ultrasound-triggered hydrogelation. We then exposed the conjugates to ultrasound for 5 s, and evaluated the suspension using bright field microscopy and an *o*-CPC calcium assay. We were unable to identify any microbubble–liposome conjugates after ultrasound exposure, indicating widespread destruction of the microbubble population (Figure 3e). Under these conditions, the microbubble–liposome conjugates liberated approximately double the amount of calcium (47 ± 8%) than dose-matched liposomes (21 ± 4%) (Figure 3f). This observation indicated that microbubble conjugation could enhance the efficiency of ultrasound-triggered liposomal calcium ion release. We next showed that we could trigger transglutaminase-catalyzed hydrogelation of fibrinogen by exposing calcium-loaded microbubble–liposome conjugates to 5 s of ultrasound (Figure 3g). At the 42 h endpoint, the ultrasound-exposed system had visibly gelled, while no gelation was observed in the unexposed control group (Figure 3h). A limitation of using microbubbles is the relatively low yield of conjugates that can be produced. As a result, this study used a lower level of total encapsulated calcium than for the liposome system, which is likely to have caused the longer hydrogelation. There is scope to increase hydrogelation kinetics, however, this would require scaled-up manufacturing processes in order to generate the necessarily high concentrations of microbubble-liposome conjugates.

In this report, we have presented a new approach to achieve ultrasound-triggered enzyme catalysis, which we used for ultrasound-triggered enzymatic hydrogelation. We have shown that a brief application of ultrasound (1–50 s) could be used to controllably liberate liposomal calcium ions, which could subsequently activate transglutaminase catalysis. We used this ultrasound-triggered catalysis to enzymatically crosslink fibrinogen and form self-supporting, viscoelastic hydrogels. Importantly, the calcium ion release, enzyme kinetics, and gelation rate could all be tuned by varying the ultrasound exposure time. We also demonstrated that calcium-loaded liposomes could be conjugated to gaseous microbubbles to enhance the liposomal payload release upon ultrasound exposure. These calcium-loaded microbubble–liposome conjugates were also used for ultrasound-triggered hydrogelation of fibrinogen. Taken together, these results suggest that ultrasound can provide an entirely new stimulus for enzyme activity and radical-free hydrogelation, alongside the traditional triggers of light, pH, temperature, and chemical addition. The use of ultrasound enables catalysis or hydrogelation to be remotely triggered at a chosen time (e.g., after components have been mixed or injected), while the enzyme kinetics, gelation rate and final hydrogel stiffness can be tailored using different ultrasound exposure times.

A major advantage of ultrasound is that it can propagate through opaque materials with much less attenuation than UV or visible light. We thus anticipate that the greatest benefit of this method will be in the ability to trigger catalysis or gelation in conventionally inaccessible scenarios found in industry (e.g., opaque containers/pipes), academia (e.g., closed microfluidic systems), and medicine (e.g., in vivo gelation). Many of these applications would require further optimization, e.g., an enzyme system with higher ion threshold would be required for in vivo applications, while the use of higher frequency focused ultrasound would enable more biocompatible and remote triggering.^[52]^ It should be noted that while transglutaminase was used as an exemplar in this work, this method is modular, with the cofactor loaded in the liposomes and the enzyme present in the surrounding solution. Thus, the exact same principles could be applied to other enzymes with ionic cofactors, which include many oxidoreductases, transferases, hydrolases, lyases, isomerases, and ligases.^[53]^ Similarly, fibrinogen was used as a proof-of-concept hydrogel, however, many other materials use ion-dependent crosslinking, such as alginate,^[54]^ pectin,^[55]^ cellulose nanofibrils,^[56]^ chitosan,^[57]^ and sodium polygalacturonate.^[58]^ This versatility enables diverse applications for this platform technology in molecular biology, synthetic biology, and material science.

## Supplementary Material

Supporting Information is available from the Wiley Online Library or from the author.

SI

## Figures and Tables

**Figure 1:**
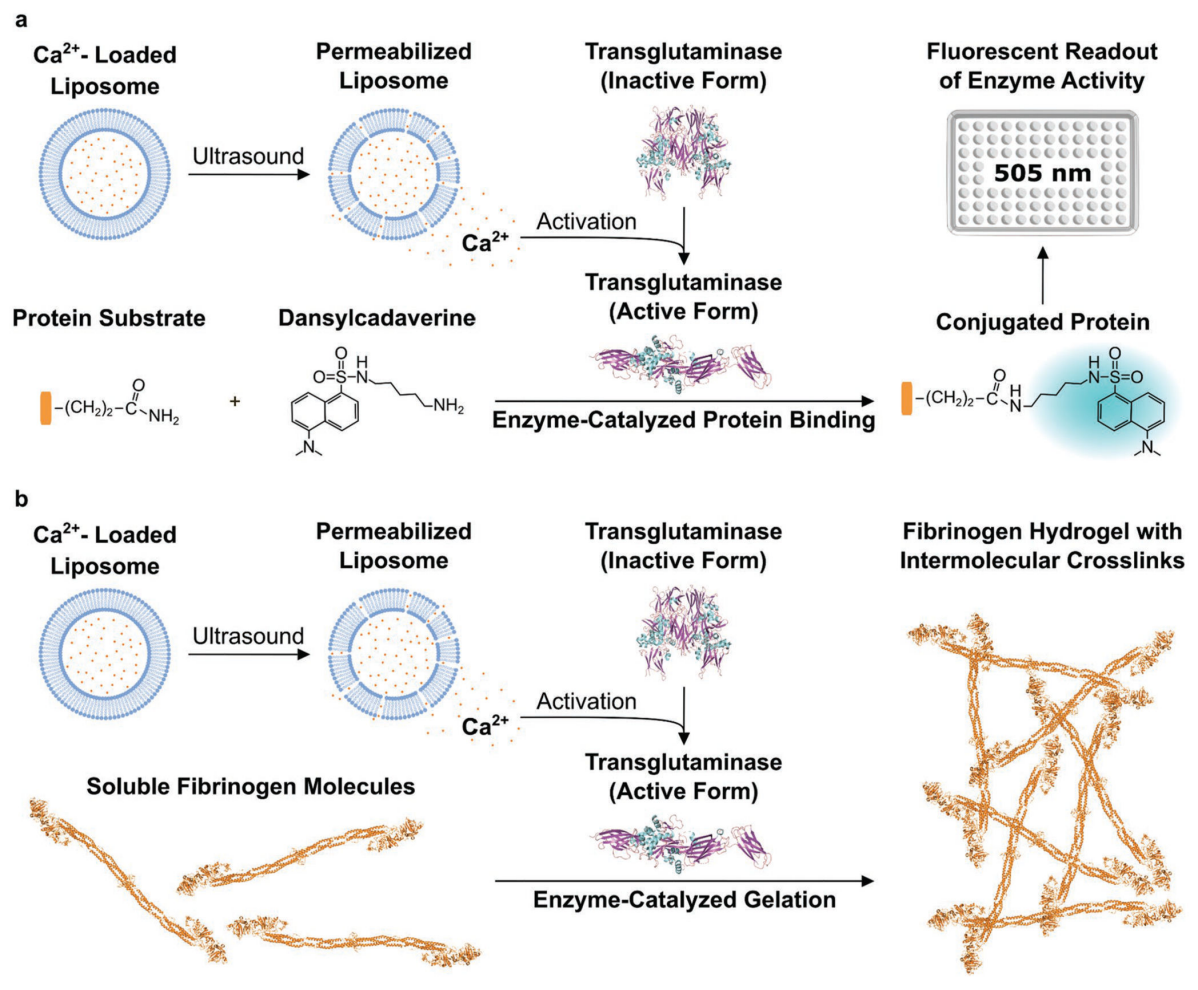
Schematic of ultrasound-triggered enzyme catalysis and hydrogelation. a) Ultrasound is applied to calcium-loaded liposomes in order to liberate calcium ions and activate transglutaminase. The active transglutaminase then catalyzes isopeptide bond formation between a protein substrate and dansylcadaverine. This conjugation produces a shift in the maximum fluorescence emission wavelength of dansylcadaverine and an increase in fluorescence intensity at 505 nm. b) A similar ultrasound-triggered process is used to catalyze the crosslinking of soluble fibrinogen molecules. In this scenario, intermolecular crosslinking is used to generate fibrinogen hydrogels. The graphics for the structures of inactive and active transglutaminase and of fibrinogen were adapted from the Protein Data Bank (PDB) and processed with Visual Molecular Dynamics (VMD) software. Inactive transglutaminase PDB ID: 1kv3; active transglutaminase PDB ID: 2q3z; fibrinogen PDB ID: 3ghg.^[30]^ The graphics for the 96-well plate were adapted from the Servier Medical Art website.

**Figure 2:**
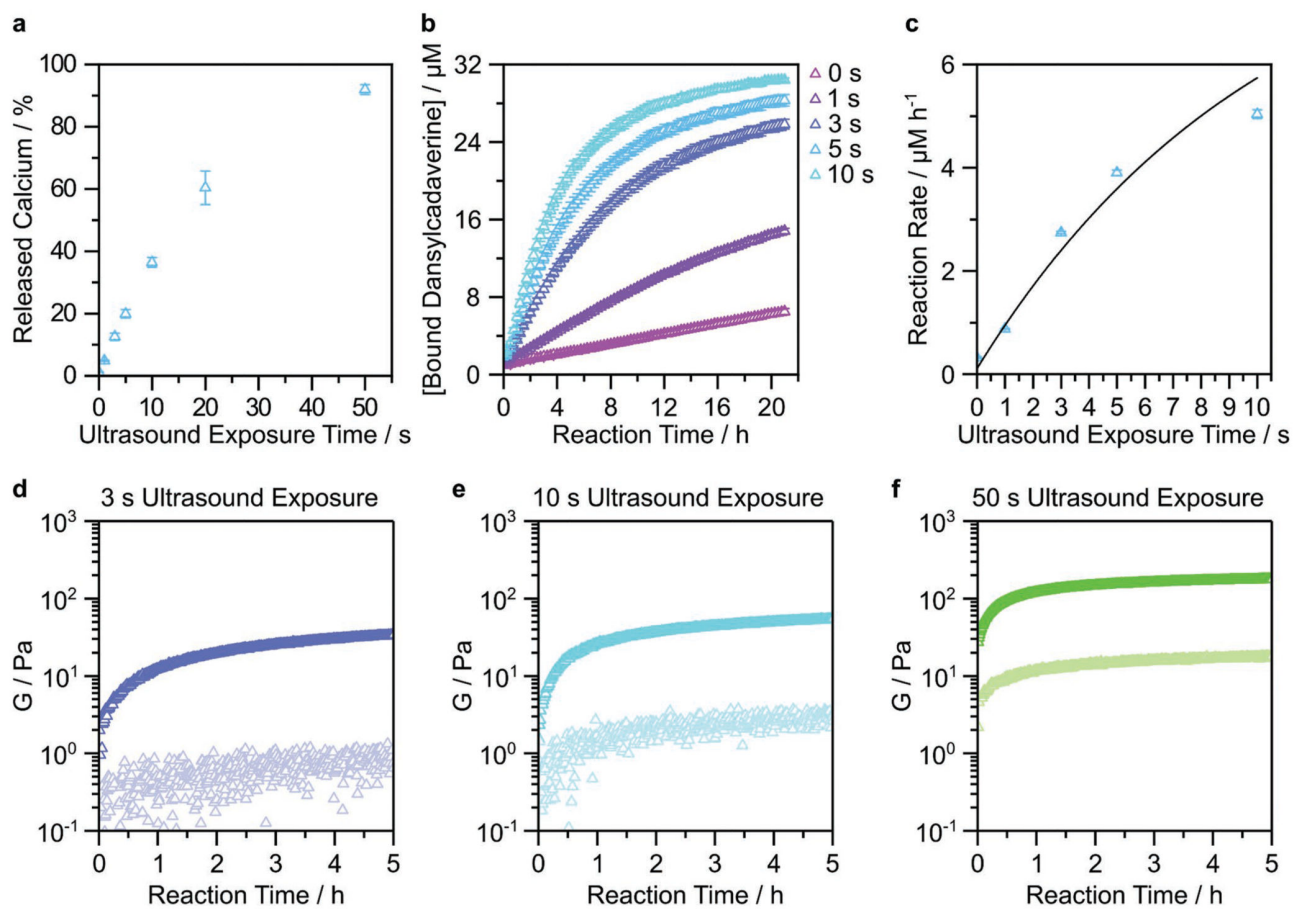
Ultrasound-triggered enzyme catalysis and hydrogelation using calcium-loaded liposomes. a) Calcium-loaded liposomes were exposed to ultrasound for 0–50 s, with the released calcium quantified using an *o*-CPC assay. Data shown are the mean and standard deviation from four technical replicates across two batches of liposomes. b) The enzymatically catalyzed conversion of dansylcadaverine was measured after calcium-loaded liposomes were exposed to ultrasound for 0–10 s. Data shown are the mean and standard deviation from three technical replicates from one batch of liposomes. c) The rate of dansylcadaverine conversion was measured as a function of ultrasound exposure. Data shown are the mean and standard deviation, fitted to an asymptotic regression model (*R*^2^ = 0.95). d–f) The transglutaminase-catalyzed hydrogelation of fibrinogen was measured using timesweep rheology after the application of ultrasound for 3 s (d), 10 s (e), or 50 s (f). The final concentrations of fibrinogen and transglutaminase were 22.4 mg mL^–1^ and 5 × 10^–6^ m, respectively. Measurements were carried out at 1% strain and 1 rad s^–1^ at 25°C. *G*′ and *G*″ are shown with dark and light markers, respectively. Data shown for one technical repeat. For unexposed controls, see Figure S8 in the Supporting Information.

**Figure 3:**
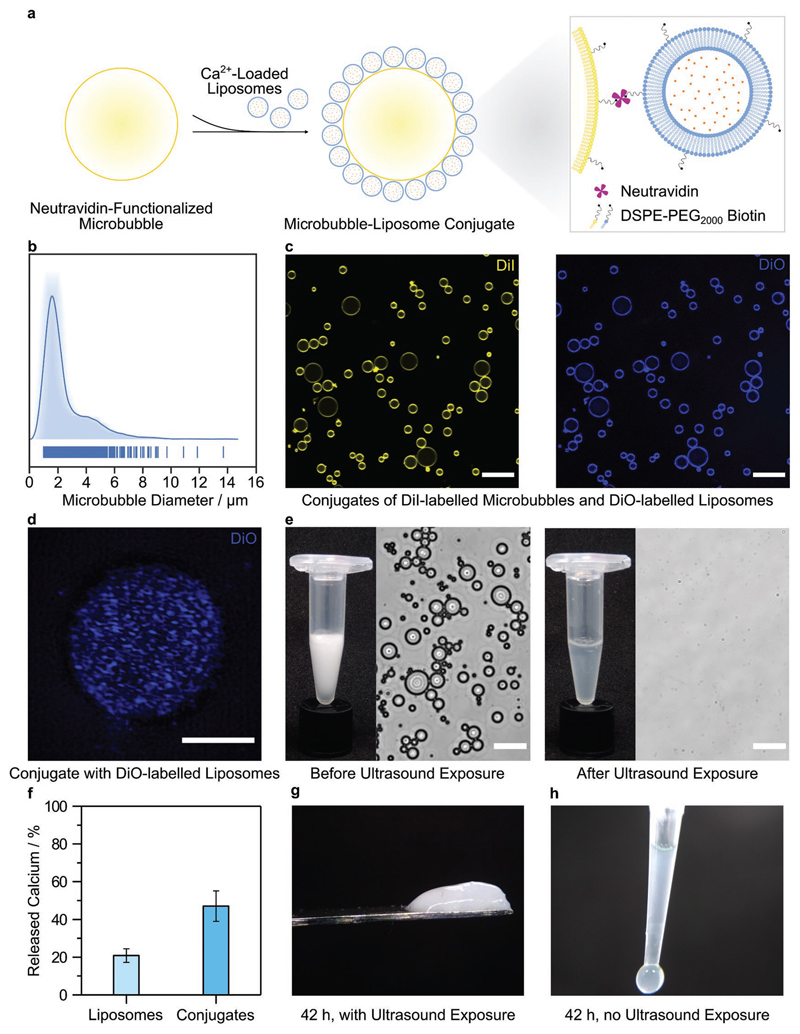
Ultrasound-triggered hydrogelation using calcium-loaded microbubble–liposome conjugates. a) Schematic of the microbubble–liposome conjugation process, not to scale. b) Average-shifted histogram showing the diameter distribution of the microbubbles, as determined by image analysis of 890 microbubbles. The average diameter was measured as 2.5 ± 1.6 μm (mean ± standard deviation). c) Confocal fluorescence microscopy showing conjugates with fluorescence from both 1,1′-dioctadecyl-3,3,3′,3′-tetramethylindocarbocyanine (DiI)-labeled microbubbles (yellow) and 3,3′-dioctadecyloxacarbocyanine perchlorate (DiO)-labeled liposomes (blue). Scale bar: 20 μm. d) Super-resolution *z*-projection of DiO-labeled liposomes (blue) conjugated to the surface of a microbubble, obtained using structured illumination microscopy. Scale bar: 3 μm. e) Camera images and bright-field microscopy showing a turbid solution of intact microbubble–liposome conjugates before ultrasound exposure and a clear solution with no observable conjugates after ultrasound exposure (20 kHz, 25% duty cycle, 20% amplitude, 5 s). Scale bar: 20 μm. f) The percentage of calcium ions released from dose-matched liposomes and microbubble–liposome conjugates after ultrasound exposure (20 kHz, 25% duty cycle, 20% amplitude, 5 s) was measured using an *o*-CPC assay. Data are shown as mean and standard deviation of six technical replicates from the same batch of sonicated liposomes or microbubble–liposome conjugates. g) Image of a fibrinogen hydrogel formed after exposing calcium-loaded microbubble–liposome conjugates to ultrasound for 5 s. h) Image of an unexposed control, which remained liquid after 42 h.
