# Supplementary material for: Ultrasound-Triggered Enzymatic Gelation
Source: Adv Mater. Author manuscript; Available in PMC 2020 Apr 23. (PMC7180077; doi:10.1002/adma.201905914)
Supplement: SI [file EMS85758-supplement-SI.docx]

Supporting Information

Ultrasound-Triggered Enzymatic Gelation

Valeria Nele, Carolyn E. Schutt, Jonathan P. Wojciechowski, Worrapong Kit-Anan, James J. Doutch, James P. K. Armstrong,^*^ & Molly M. Stevens^*^

V. Nele, Dr. C. E. Schutt, Dr. J. P. Wojciechowski, Dr. W. Kit-Anan, Dr. J. P. K. Armstrong, Prof. M. M. Stevens

Department of Materials, Department of Bioengineering and Institute of Biomedical Engineering, Imperial College London, Prince Consort Road, London, SW7 2AZ, United Kingdom

Dr. C. E. Schutt

Current address: Department of Biomedical Engineering and Knight Cancer Institute's Cancer Early Detection Advanced Research Center (CEDAR), Oregon Health and Science University, Portland, Oregon, United States of America

Dr. J. J. Doutch

ISIS Neutron and Muon Source, STFC, Rutherford Appleton Laboratory, Didcot, OX11 ODE, United Kingdom.

* Corresponding author email addresses:

[m.stevens@imperial.ac.uk](mailto:m.stevens@imperial.ac.uk), james.armstrong@imperial.ac.uk

**Supplementary Experimental Section**

*Materials*

1,2-dipalmitoyl-*sn*-glycero-3-phosphocholine (DPPC), 1,2-distearoyl-*sn*-glycero-3-phosphoethanolamine-*N*-[biotinyl(polyethylene glycol)-2000] (DSPE-PEG_2000_ biotin), 1,2-distearoyl-*sn*-glycero-3-phosphocholine (DSPC) and 1,2-distearoyl-*sn*-glycero-3-phosphoethanolamine-*N*-[methoxy(polyethylene glycol)-2000] (ammonium salt) (DSPE-PEG_2000_) were purchased from Avanti Polar Lipids. All other reagents were purchased from Sigma Aldrich. Ultrapure water (18.2 MΩ cm) was taken from TR Duo10 UF Polisher triple (Triple Red, Avidity Science).

*Liposome Formulation*

Calcium-loaded liposomes were formulated using an established interdigitation-fusion vesicle method.^[1]^ Briefly, a solution of 99 mol% DPPC and 1 mol% DSPE-PEG_2000_ biotin was prepared in chloroform, dried with a stream of nitrogen gas in a glass vial and then kept under vacuum for at least 3 h. The lipid film was hydrated to a lipid concentration of 20 mg mL^-1^ with an aqueous CaCl_2_ solution for 1 h at 55 °C under constant stirring. The liposome solution was extruded 25 times through a 100 nm polycarbonate membrane and 31 times through a 50 nm polycarbonate membrane (Whatman® Nucleopore Track-Etched™ membranes) at 55 °C. To induce interdigitation, ethanol was added to a final concentration of 4 M while stirring. The interdigitated gels were stored overnight at 4 °C. Five centrifuge washes at 8000 g for 8 min were performed to remove the ethanol, after which the lipid gels were incubated at 55 °C for 2.5 h to form large unilamellar liposomes. These liposomes were then extruded 31 times through a 400 nm polycarbonate membrane (Whatman® Nucleopore Track-Etched™ membranes) at 55 °C to yield a monodisperse population of unilamellar vesicles. The calcium-loaded liposomes were dialyzed against iso-osmotic buffer (0.6 M NaCl) to remove free calcium, and then stored at 4 °C prior to use.

*Small-Angle Neutron Scattering (SANS)*

SANS measurements were performed at the SANS2D beamline of the ISIS pulsed neutron source at the Rutherford Appleton Laboratory (Didcot, UK). Samples were loaded in 1 mm path length quartz cuvette cells and measured at 25 °C. The source-to-sample (*L_1_*) and sample-to-detector (*L_2_*) distances were set as *L_1_* = *L_2_* = 4 m to give a scattering vector (*Q*) range 0.004 to 0.722 Å^-1^. The scattering angle (*θ*) was measured for neutrons of wavelengths (*λ* = 1.75-16.5 Å) used simultaneously by time-of-flight. ***Q*** has a modulus of:

$$Q= \frac{4\pi}{\lambda}\sin\left( \frac{\theta}{2} \right) \left( 1 \right).$$

Data was reduced using MantidPlot^[2]^ and the SANS curves were fitted with SasView v4.1.0^[3]^ using a Lamellar Model. This model describes a lyotropic lamellar phase with uniform scattering length density and random distribution. The 1D scattered intensity $I(Q)$ is:

$$I\left( Q \right)= \varphi* \frac{2\pi*P\left( Q \right)}{Q^{2}*\delta}+bkg \left( 2 \right),$$

where $\varphi$ is a scale factor, $Q$ is the modulus of the scattering vector, $\delta$ is the total layer thickness and $P\left( Q \right)$ is the form factor, defined as:

$$P\left( Q \right)= \frac{2\Delta\rho^{2}}{Q^{2}} \left( 1-\cos\left( Q\delta\right) \right) \left( 3 \right).$$

In this case $\Delta\rho$ is the scattering length density difference. A Gaussian polydispersity function of 15% was used for the bilayer thickness to account for the presence of the PEGylated lipid.

*Cryogenic Transmission Electron Microscopy (Cryo-TEM)*

Liposome samples for cryo-TEM were prepared using an automatic plunge freezer (Leica EM GP). Briefly, 4 µL of sample was deposited on QuantiFoil R2/1 copper grids (Electron Microscopy Supplies) in an environmentally-controlled chamber at 90% relative humidity and 20 °C. Prior to deposition, the grids were plasma treated (O_2_/H_2_ 1:1 for 15 s) using a Gatan SOLARIS plasma cleaner.

After blotting the excess suspension on filter paper, the sample was vitrified in liquid ethane. Samples were stored in liquid nitrogen and imaged at -170 °C using a Gatan 914 cryo-holder in a JEOL 2100Plus transmission electron microscope at 200 kV. Minimum Dose System software was used for imaging, with micrographs acquired using a Gatan Orius SC 1000 camera with a 5 s exposure time, a magnification of 30000 or 15000 and no image binning.

*Liposome Sizing and Quantitation*

Samples were prepared for dynamic light scattering (DLS) by dilution to 1.2 × 10^12^ particles mL^-1^ in iso-osmotic buffer. Measurements were made using a Zetasizer Nano ZS (Malvern), with normalized intensity, volume and number distribution reported as a function of the hydrodynamic diameter. Nanoparticle tracking analysis (NTA) measurements were performed using samples diluted to a concentration of 10^8^ - 10^9^ particles mL^-1^ in iso-osmotic buffer. Three 60-s videos were acquired using a NanoSight NS300 at a camera level of 13 and analyzed using NTA V3.0 software with a detection threshold of 5.

*Quantifying Calcium Loading into Liposomes*

Liposomes were formulated with either 0.2, 0.4 or 0.6 M aqueous CaCl_2_ solutions, as described above. In order to quantify the total encapsulated calcium, the liposomes were lyzed with 5 vol% Triton X-100 at 55 °C for 40 min under stirring and an *o*-cresophtalein complexone (*o*-CPC) assay was then performed. 24.4 µL of each sample was mixed with 24.4 µL of 0.1 M HCl and 132.2 µL of a solution containing 10 mg mL^-1^ of *o*-CPC in sodium borate buffer (prepared by adding 2 M NaOH to an aqueous solution of 250 mM boric acid to yield a final pH of 10). The absorbance at 570 nm was measured in a black clear-bottom 96-well half-area plate using a SpectraMax M5 microplate reader. Nanoparticle tracking analysis was used to measure the liposome concentration (see above for full details), which was used to normalize the measured total encapsulated calcium.

*Quantifying Liposomal Calcium Leakage*

Liposomes prepared with 0.4 M CaCl_2_ solution were incubated in a 0.6 M NaCl solution at 25 °C over 5 d. Aliquots were taken at different time points and an *o*-CPC assay performed to measure the concentration of free calcium. In order to be within the linear range of the *o*-CPC assay, the liposomes were diluted to a total encapsulated calcium concentration of 2.55 mM prior to the experiment. A standard curve of CaCl_2_ and calcium-free liposomes encapsulating 0.6M NaCl, at the same particle concentration of the calcium-loaded liposomes, was used to calculate the concentration of calcium in the unknown samples.

*Ultrasound-Triggered Calcium Release from Liposomes*

Ultrasound was applied with a probe sonicator (VibraCell VC 750 with 2 mm diameter microtip, Sonics & Materials Inc.) using 20 kHz, 20% amplitude and 25% duty cycle. These parameters were used for all ultrasound-triggered studies. Ultrasound was applied to 250 μL of calcium-loaded liposomes in a 500 μL LoBind DNA Eppendorf tube for 1, 3, 5, 10 or 20 s. For the 50 s exposure, two 25 s ultrasound applications were used with a 40 s interval. An *o*-CPC assay was performed to quantify the concentration of released calcium. In order to be within the linear range of the *o*-CPC assay, the liposomes were diluted to a total encapsulated calcium concentration of 2 mM prior to the experiment. A standard curve of free CaCl_2_ in 0.6M NaCl was used to calculate the quantity of calcium in the unknown samples. Ultrasound-triggered calcium release from liposomes was also tested immediately after formulation and 5 d post formulation. In this case, ultrasound was applied to 250 μL of calcium-loaded liposomes in a 500 μL LoBind DNA Eppendorf tube for 50 s (two 25 s ultrasound applications with a 40 s interval). An *o*-CPC assay was performed to quantify the concentration of released calcium, as described above.

*Ultrasound-Triggered Catalysis*

Calcium-loaded liposomes were diluted in order to have a total encapsulated calcium concentration of 1 mM. 250 μL aliquots were transferred to a 500 μL LoBind DNA Eppendorf tube, and ultrasound was applied as previously described. Transglutaminase activity was assessed with a dansylcadaverine-based assay. An assay solution was made using dansylcadaverine in 50 mM TRIS-HCl buffer and 25 vol% DMSO, *N,N*-dimethylcasein, DTT and liposomes sonicated for 0, 1, 3, 5, 10 with a probe sonicator. The final concentrations of dansylcadaverine, *N,N*-dimethylcasein and DTT were 47.7 μM, 0.298 mg mL^-1^ and 2.98 mM, respectively. 7.86 μL of 1.91 μM aqueous transglutaminase solution was added to 142.2 μL of assay mixture in a black clear bottom 96-well half-area plate. The final concentration of transglutaminase was 100 nM. Fluorescence intensity was measured using a SpectraMax M5 microplate reader (ex: 360 nm, em: 505 nm, bottom read) over 21 h, with a cover film used to prevent sample evaporation. For endpoint measurements, ultrasound was applied for 0, 1, 3 or 5 s to a mixture of assay solution and transglutaminase at the same ratios as previously described. Samples were then transferred in a black clear bottom 96-well half-area plate (150 μL/well), covered with a PCR cover film and incubated for 21 h, before measuring the fluorescence intensity using a SpectraMax M5 microplate reader (ex: 360 nm, em: 505 nm, bottom read). All the experiments were performed at 25 °C.

*Enzyme Kinetics*

A standard curve was used to convert the fluorescence intensity into the concentration of reacted dansylcadaverine. An assay solution was made using dansylcadaverine in 50 mM TRIS-HCl buffer and 25 vol% DMSO, *N,N*-dimethylcasein and DTT. The final concentrations of dansylcadaverine, *N,N*-dimethylcasein and DTT were 47.7 μM, 0.298 mg mL^-1^ and 2.98 mM, respectively. A solution of calcium chloride in 0.6 M NaCl was then added to the mixture to a final concentration of 1 mM, together with transglutaminase to a final concentration of 100 nM. Control samples were prepared by adding an equivalent volume of deionized water in place of the transglutaminase or an equivalent volume of 0.6 M NaCl in place of the calcium chloride solution. After 43 h incubation, a standard curve was prepared by mixing ratios of transglutaminase-containing samples and negative control samples. The fluorescence intensity was measured in a black clear bottom 96-well half-area plate using a SpectraMax M5 microplate reader (ex: 360 nm, em: 505nm). This enabled the concentration of bound dansylcadaverine to be plotted as a function of time for the ultrasound-triggered catalysis. Using these graphs, the gradient of the linear portion of the curves (up to 3 h) were measured and plotted as a function of the ultrasound exposure time. This data was fitted with an asymptotic nonlinear regression model (R^2^ = 0.94) using OriginPro 2017 software.

*Effect of Ultrasound on Enzyme Activity*

A mixture containing transglutaminase and calcium-loaded liposomes was exposed to ultrasound for 3, 10 or 50 s. The substrate mixture (dimethylcasein and dansylcadaverine) was added at different time points post-exposure (0, 24, 48 and 72 h) and the fluorescence intensity at 505 nm was recorded after 24 h incubation with the substrate. As a control, calcium-loaded liposomes were exposed to ultrasound for 3, 10 and 50 s and mixed with unexposed transglutaminase and the substrate mixture at the corresponding time points. The final concentrations of dansylcadaverine, *N,N*-dimethylcasein and DTT were 47.7 μM, 0.298 mg mL^-1^ and 2.98 mM, respectively. The final concentration of transglutaminase was 100 nM. A final volume of 75 µL of mixture was transferred to a black clear bottom 96-well half-area plate prior to the fluorescence measurement. A standard curve for dansylcadaverine was prepared, as described previously but with a final volume of 75 µL.

*Ultrasound-Triggered Hydrogelation using Calcium-Loaded Liposomes*

250 μL of calcium-loaded liposomes (total encapsulated calcium of 53.6 mM) was transferred to a 500 μL LoBind DNA Eppendorf tube and sonicated for 0, 3, 10 or 50 s with a probe sonicator. 100 µL of each liposome group were then mixed with DTT in deionized water (final DTT concentration of 8.69 mM) and fibrinogen in 0.6 M NaCl (final fibrinogen concentration of 22.42 mg mL^-1^). Transglutaminase was then added to a final concentration of 5 μM immediately prior to rheological measurements. A time sweep was performed over 5 h at 1% strain and 1 rad s^-1^ with an AR 2000 rheometer (TA instruments) equipped with an 8 mm steel parallel plate and an oil chamber to prevent solvent evaporation. The unexposed group was characterized using frequency and strain sweeps. The frequency sweep measurements (0.1 to 100 rad s^-1^) were performed at 1% strain while the strain sweep measurements (0.1 to 100% strain) were performed at 1 rad s^-1^. All experiments were performed at 25 °C. The same protocol was followed for the ultrasound-triggered gelation experiment with varying transglutaminase concentration. In this case, transglutaminase was then added to a final concentration of 1.25, 5 or 10 μM immediately prior to rheological measurements. A time sweep was performed over 3 h at 1% strain and 1 rad s^-1^ with an AR 2000 rheometer (TA instruments) equipped with an 8 mm steel parallel plate and an oil chamber to prevent solvent evaporation.

*Microbubble Formulation and Sizing*

Microbubbles were formulated using a method adapted from a previously reported protocol.^[4]^ A lipid film comprising 1,2-distearoyl-*sn*-glycero-3-phosphocholine (DSPC), DSPE-PEG_2000_ and DSPE-PEG_2000_ biotin in an 86:9:5 molar ratio was hydrated with 0.6 M NaCl to a final lipid concentration of 6.32 mg mL^-1^. The lipid suspension was vortexed for 15 s and heated at 75 °C for 2 min, then vortexed and heated once more, ending with an additional 15 s vortex. A perfluorohexane/air mixture was pumped over the lipid suspension and the sample was sonicated using a VibraCell VC 750 with 2 mm diameter microtip (Sonics & Materials Inc.) probe sonicator (20 kHz, 40% amplitude, 100% duty cycle, 3 s). Four centrifuge washes (100 g, 3 min) were performed to remove excess lipid. Samples were imaged on an Olympus IX71 inverted microscope in bright field mode with a 60X oil immersion objective lens. Automated image analysis was performed using ImageJ for microbubble sizing. Briefly, the automated threshold and fill holes functions in ImageJ were applied to the image; an adjustable watershed function with a 0.4 tolerance was used. Automated particle sizing was carried out on the microbubbles by specifying a minimum pixel size and selecting a 0.8 – 1.0 circularity interval. The average-shifted histogram was generated *via* the Buriak group data plotter website (<https://maverick.chem.ualberta.ca/plot/ash>).

*Microbubble-Liposome Conjugation*

To form the conjugates, 400 µL biotinylated microbubbles were incubated with 21 µL of an aqueous 10 mg mL^-1^ neutravidin solution for 15 min at 300 rpm and 22 °C in an Eppendorf Thermomixer Comfort. Four centrifuge washes were performed (100 g, 3 min) to remove any unbound neutravidin. 200 µL of neutravidin-functionalized microbubbles were then incubated with 200 µL of calcium-loaded liposomes for 30 min at 300 rpm and 22 °C in an Eppendorf Thermomixer Comfort. The mixture was prepared with 7 × 10^5^ liposomes per microbubble. Four centrifuge washes were performed (100 g, 3 min) to remove unbound liposomes. Microbubble-liposome conjugates were also imaged on a Leica SP5 inverted confocal fluorescence microscope in bright field and fluorescence mode with a 63X oil immersion objective lens. For this experiment, microbubble-liposome conjugates were prepared using DiO-labelled liposomes and DiI-labelled microbubbles.

*Structured Illumination Microscopy (SIM)*

Conjugates were prepared using DiO-labelled liposomes and unlabelled microbubbles, then diluted in glycerol to a concentration of 6 × 10^6^ conjugates mL^-1^. 5 µL of this suspension was placed on a glass slide, covered with a coverslip and left to settle for 10 min before imaging. Micrographs were obtained on a Zeiss Elyra PS.1 microscope (Carl Zeiss) equipped with sCMOS PCO Edge using a Plan-Apochromat 63 × 1.4 NA oil-immersion DIC objective lens. Each image was recorded with three orientation angles of the excitation grid and five phases acquired for each image with a 110 nm z-step and a pixel size of 32 nm imaged at 8 bits per pixel with no image averaging. A 488 nm laser was used for imaging. SIM processing was performed using SIM module of the Zen software package (Carl Zeiss) while 3D SIM reconstruction was performed with Fiji ImageJ software (NIH).

*Ultrasound-Triggered Calcium Release from Microbubble-Liposome Conjugates*

An *o*-CPC assay was used to quantify the total encapsulated calcium level of lysed liposome and microbubble-liposome conjugate suspensions. The remaining liposome and conjugate suspensions were then diluted to a total encapsulated calcium concentration of 100 µM. These dose-matched samples were then aliquoted, with 250 µL transferred into 500 µL DNA LoBind tubes. Ultrasound was applied for 5 s with a probe sonicator, before the quantity of released calcium was measured using a second *o*-CPC assay. Conjugates were also imaged with a camera and a bright field microscope (Olympus IX71) before and after ultrasound exposure.

*Ultrasound-Triggered Enzymatic Hydrogelation from Microbubble-Liposome Conjugates*

125 µL of microbubble-liposome conjugates (total encapsulated calcium of 420 µM) were transferred into 500 µL DNA LoBind tubes. Ultrasound was applied for 5 s with a probe sonicator and a negative control was left without ultrasound exposure. 100 µL of each suspension was added to separate solutions of fibrinogen in 0.6 M NaCl (final fibrinogen concentration of 22.68 mg mL^-1^) and aqueous DTT (final DTT concentration of 10 mM) in a 500 µL Protein LoBind tube. Transglutaminase was added to a final concentration of 5 µM and samples were incubated at 25 °C for 42 h.

**Supplementary References**

[1] P. L. Ahl, L. Chen, W. R. Perkins, S. R. Minchey, L. T. Boni, T. F. Taraschi, A. S. Janoff, *Biochim. Biophys. Acta - Biomembr.* **1994**, *1195*, 237.

[2] O. Arnold, J. C. Bilheux, J. M. Borreguero, A. Buts, S. I. Campbell, L. Chapon, M. Doucet, N. Draper, R. Ferraz Leal, M. A. Gigg, V. E. Lynch, A. Markvardsen, D. J. Mikkelson, R. L. Mikkelson, R. Miller, K. Palmen, P. Parker, G. Passos, T. G. Perring, P. F. Peterson, S. Ren, M. A. Reuter, A. T. Savici, J. W. Taylor, R. J. Taylor, R. Tolchenov, W. Zhou, J. Zikovsky, *Nucl. Instruments Methods Phys. Res. Sect. A Accel. Spectrometers, Detect. Assoc. Equip.* **2014**, *764*, 156.

[3] http://www.sasview.org/. Accessed October 2018.

[4] C. E. Schutt, S. D. Ibsen, M. J. Benchimol, M. J. Hsu, S. C. Esener, *Small* **2014**, *10*, 3316.

**Supplementary Figures**

**Figure S1.** SANS analysis of DPPC / DSPE-PEG_2000_ biotin liposomes loaded using 0.4 M calcium chloride. (a) Unextruded and (b) extruded liposomes were analyzed using SANS (blue markers) and fitted to a lamellar model (black line). This analysis was used to calculate membrane bilayer thicknesses of 49.1 ± 0.1 and 50.9 ± 0.1 Å for the unextruded and extruded liposomes, respectively. Data are plotted on a log-log scale.

**Figure S2.** Representative cryo-TEM images of DPPC / DSPE-PEG_2000_ biotin liposomes loaded using 0.4 M calcium chloride. (a) Unextruded and (b) extruded liposomes were imaged using cryo-TEM, which revealed that the liposomes were unilamellar both before and after extrusion. Scale bars: 200 nm.

**Figure S3.** Sizing analysis of DPPC / DSPE-PEG_2000_ biotin liposomes loaded using 0.4 M calcium chloride. (a) DLS measurements showed single peaks for number (light blue), volume (blue) and intensity (dark blue) distributions, with a z-average hydrodynamic diameter of 122 ± 43 nm and a polydispersity of 0.125. (b) NTA measurements also showed a narrow size distribution, with an average hydrodynamic diameter of 144 ± 51 nm.


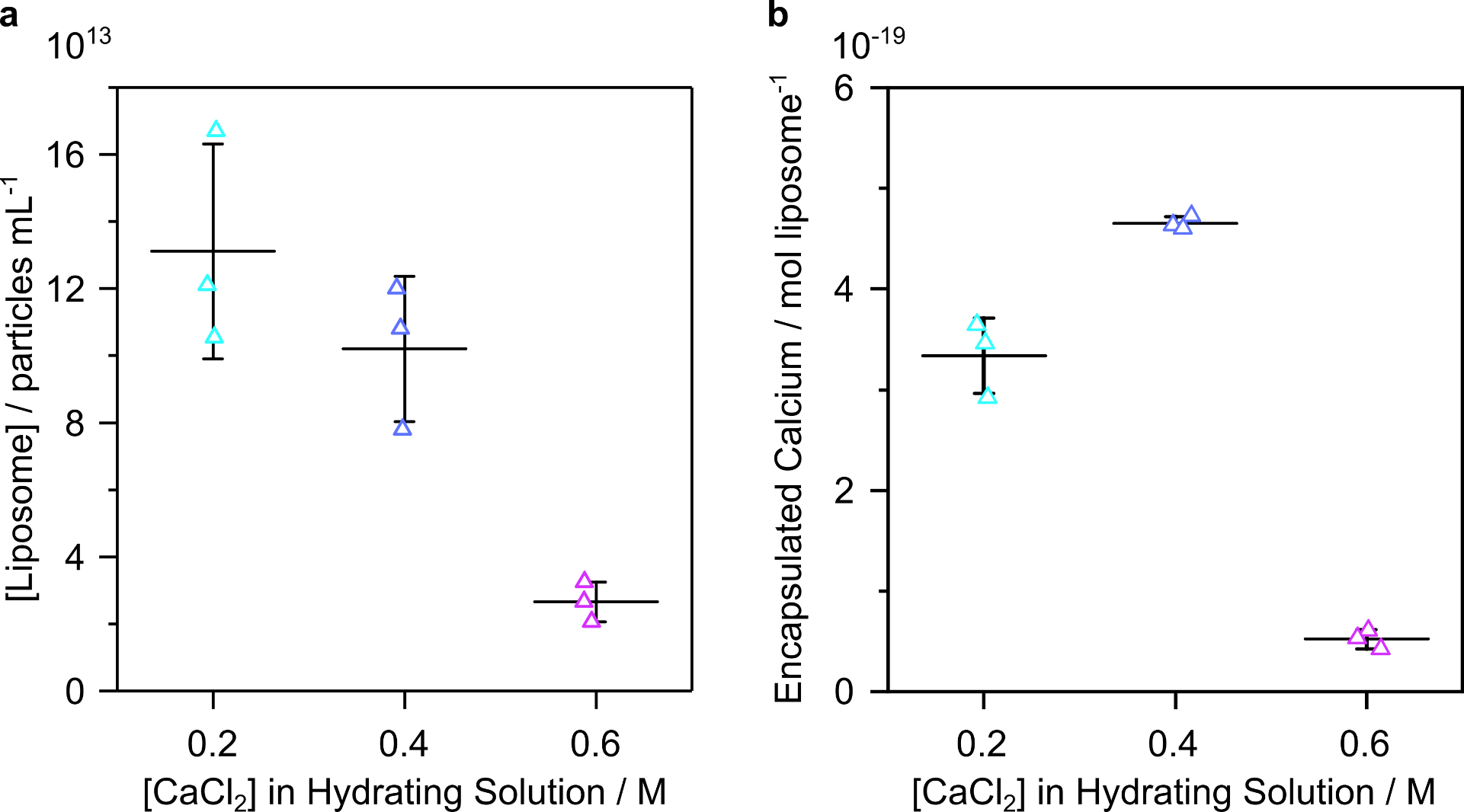


**Figure S4.** The effect of CaCl_2_ concentration during lipid hydration. (a) NTA particle counting was used to measure the yield of liposomes hydrated using different CaCl_2_ solutions. (b) An o-CPC assay was used to quantify the calcium loading into liposomes hydrated using different CaCl_2_ solutions, with this value normalized by the number of liposomes. Data shown as mean and standard deviation, with data collected from three different liposome batches.


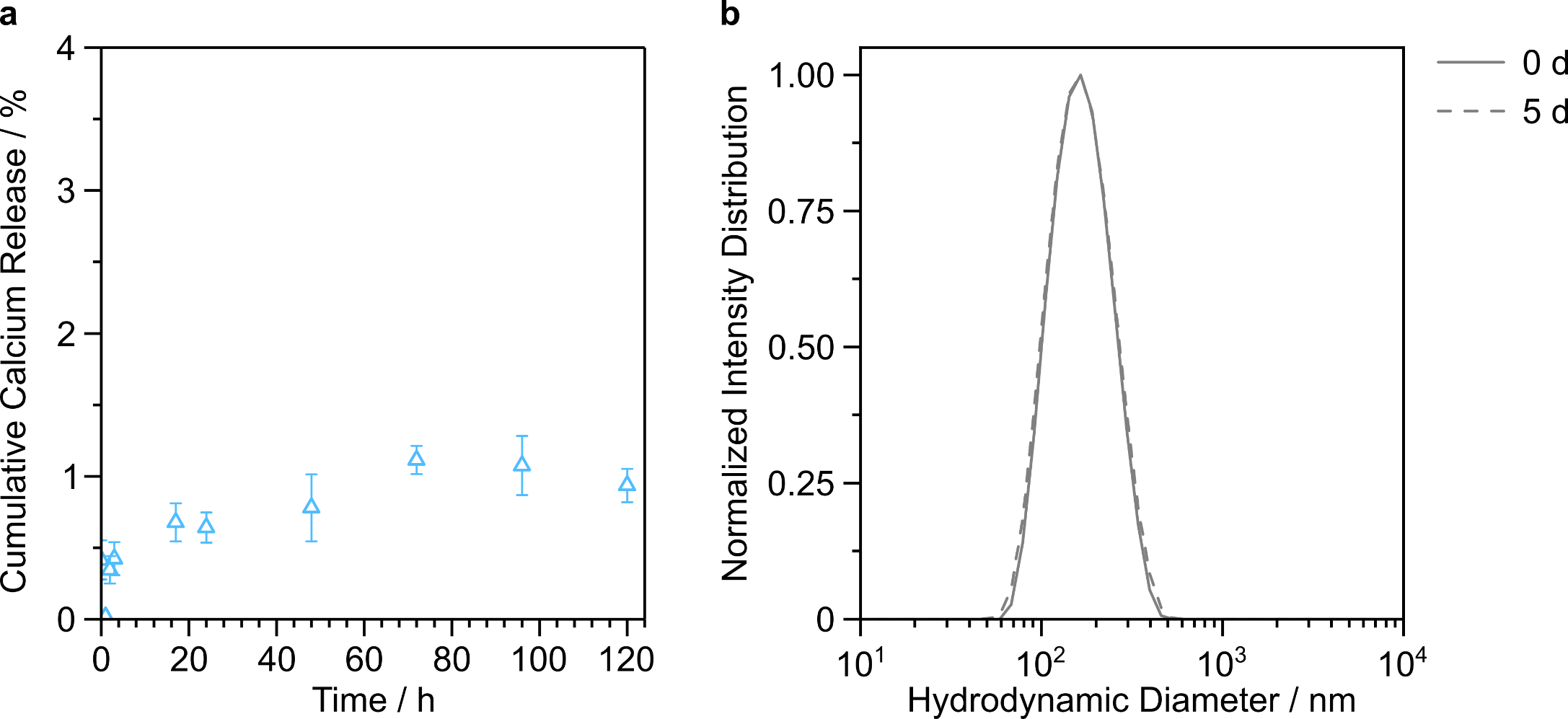


**Figure S5.** (a) Calcium leakage from DPPC / DSPE-PEG_2000_ biotin liposomes loaded using 0.4 M calcium chloride. Calcium-loaded liposomes were incubated at 25 °C for 5 d, with the released calcium measured at intervals using an o-CPC assay (blue markers). The percentage release was calculated by normalizing the values at each interval to the total calcium level measured from a lysed liposome control. Data shown as mean and standard deviation for three technical replicates using the same batch of liposomes. (b) DLS measurements were performed immediately after formulation (solid line) or after 5 d (dashed line). In both cases, a single peak for intensity distribution was observed, with z-average hydrodynamic diameters of 156 nm and 155 nm and PDI of 0.12 and 0.14, respectively.


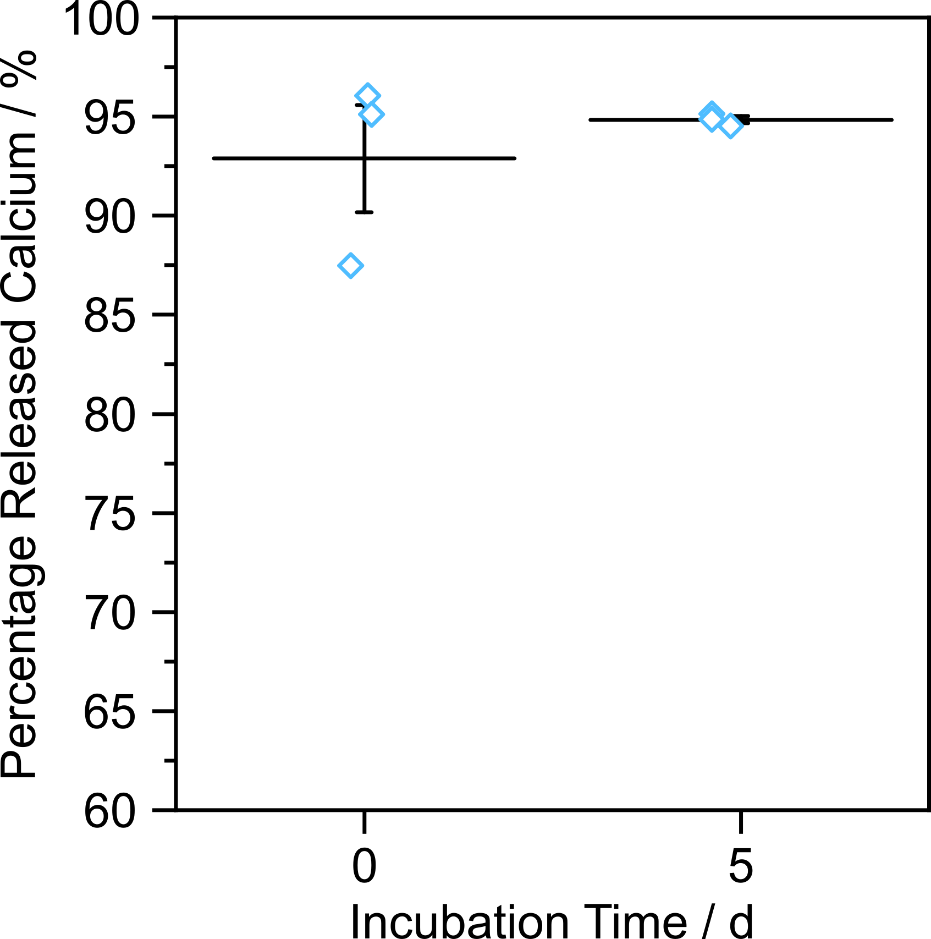


**Figure S6.** Calcium-loaded liposomes were exposed to ultrasound for 50 s immediately after formulation or after 5 days of incubation. The amount of released calcium was measured with an o-CPC assay.


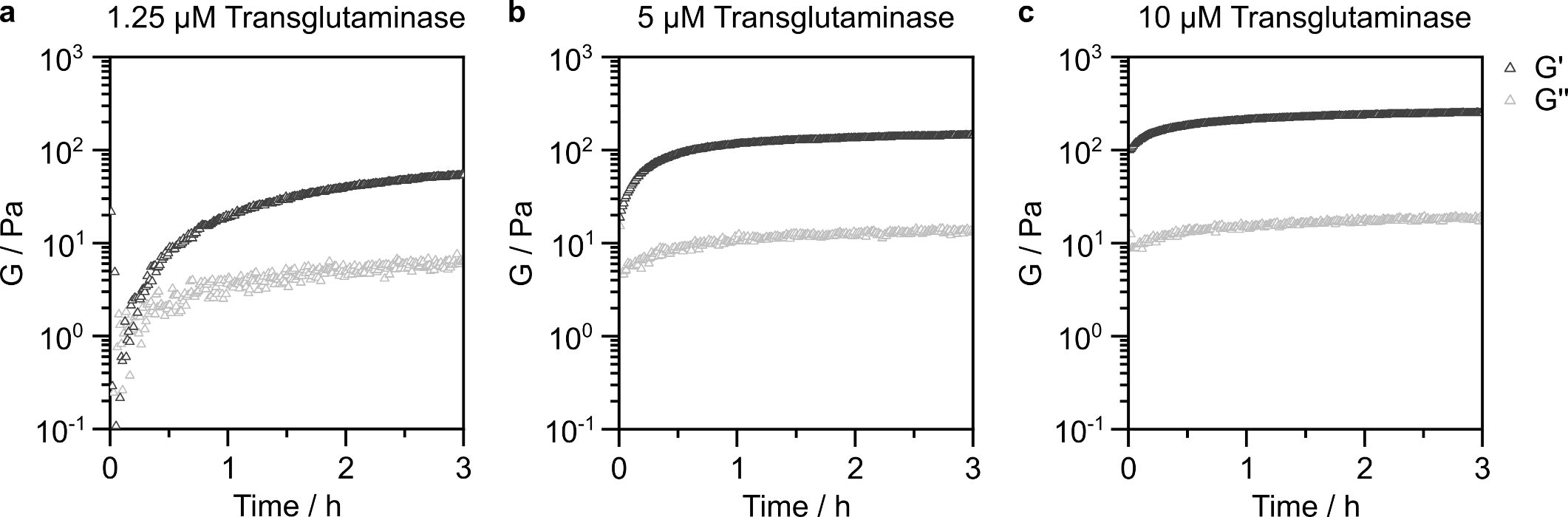


**Figure S7.** Ultrasound-triggered gelation kinetics can be tuned by changing the concentration of transglutaminase. Calcium-loaded liposomes were exposed to ultrasound for 50 s and the gelation of fibrinogen was measured using time-sweep rheometry upon the addition of (a) 1.25 µM, (b) 5 µM and (c) 10 µM transglutaminase. An increase in the gelation kinetics was observed with increasing transglutaminase concentration. Measurements were carried out at 1% strain and 1 rad s^-1^ at 25 °C.

**Figure S8.** Rheology control experiment for liposomes with no ultrasound exposure. (a) Frequency and (b) strain sweeps were performed on solutions of calcium-loaded liposomes, transglutaminase and fibrinogen that had not been exposed to ultrasound (measured after 6 h). This analysis showed these negative controls to be in liquid form, with the elastic modulus (G′, black markers) not exceeding the viscous modulus (G′′, gray markers). The frequency sweep was performed at 1% strain while the strain sweep was performed at 1 rad s^-1^ frequency. Both measurements were performed at 25 °C. Other than the lack of ultrasound exposure, all parameters used were identical to the ultrasound-triggered gelation shown in Figure 2e-f.


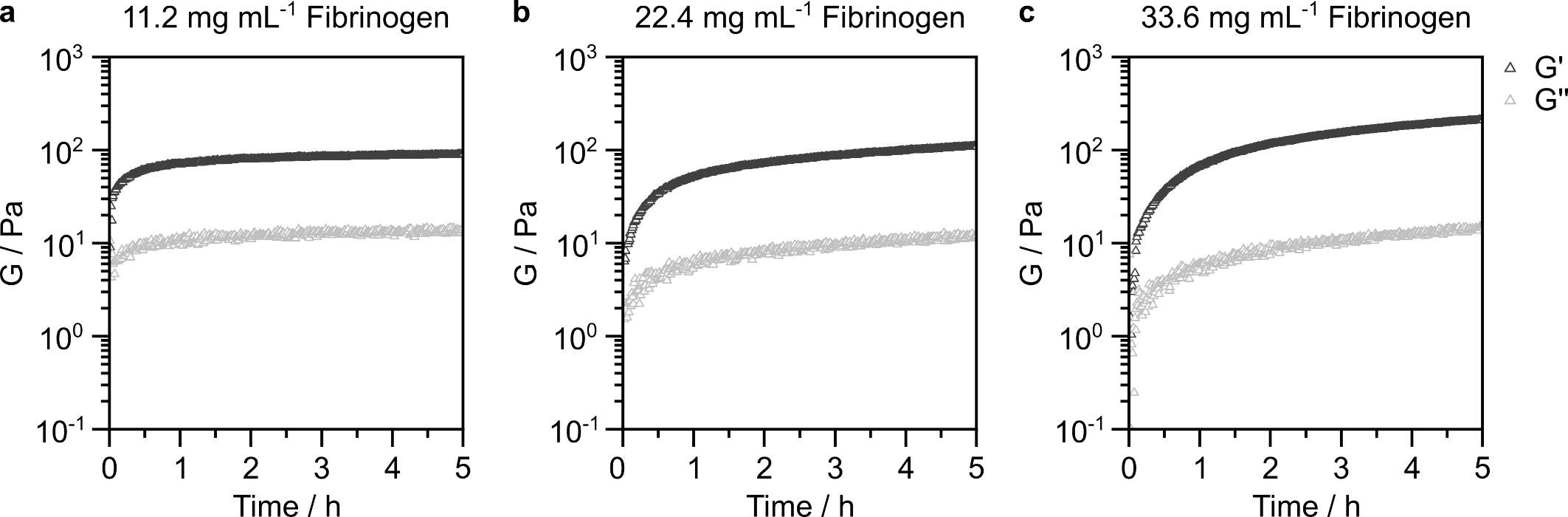


**Figure S9.** Ultrasound-triggered hydrogelation with varying fibrinogen concentration. The transglutaminase-catalyzed fibrinogen gelation upon ultrasound exposure was measured using time sweep rheology after the application of ultrasound for 50 s to calcium-loaded liposomes. After 5 h, the elastic moduli were measured as 90, 110 and 211 Pa for (a) 11.2 mg mL^-1^, (b) 22.4 mg mL^-1^ and (c) 33.6 mg mL^-1^ fibrinogen, respectively. Measurements were carried out at 1% strain and 1 rad s^-1^ at 25 °C.


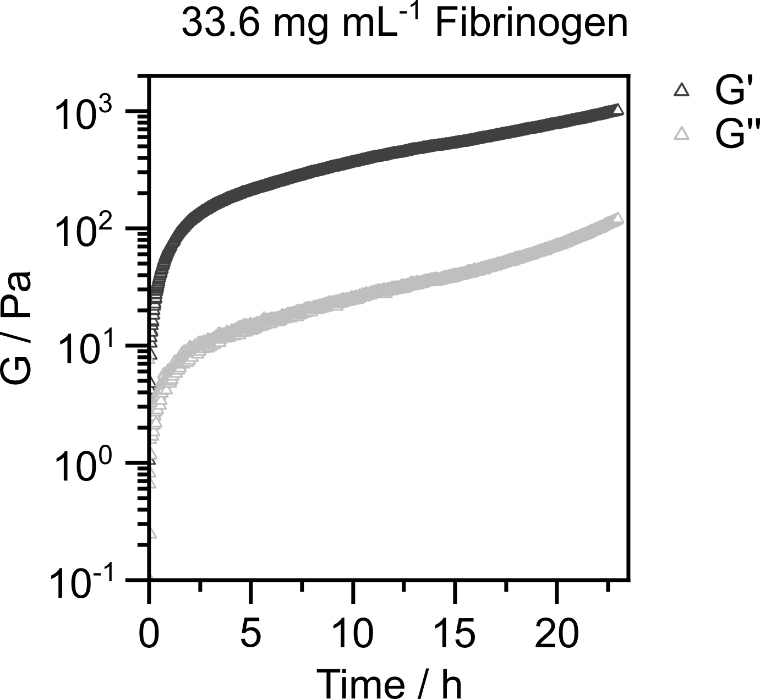


**Figure S10.** Ultrasound-triggered hydrogelation with increased crosslinking time using a 33.6 mg mL^-1^ fibrinogen solution. The transglutaminase-catalyzed fibrinogen gelation upon ultrasound exposure was measured using time sweep rheology after the application of ultrasound for 50 s to calcium-loaded liposomes. After 23 h, the elastic modulus was 1009 Pa. Measurements were carried out at 1% strain and 1 rad s^-1^ at 25 °C.


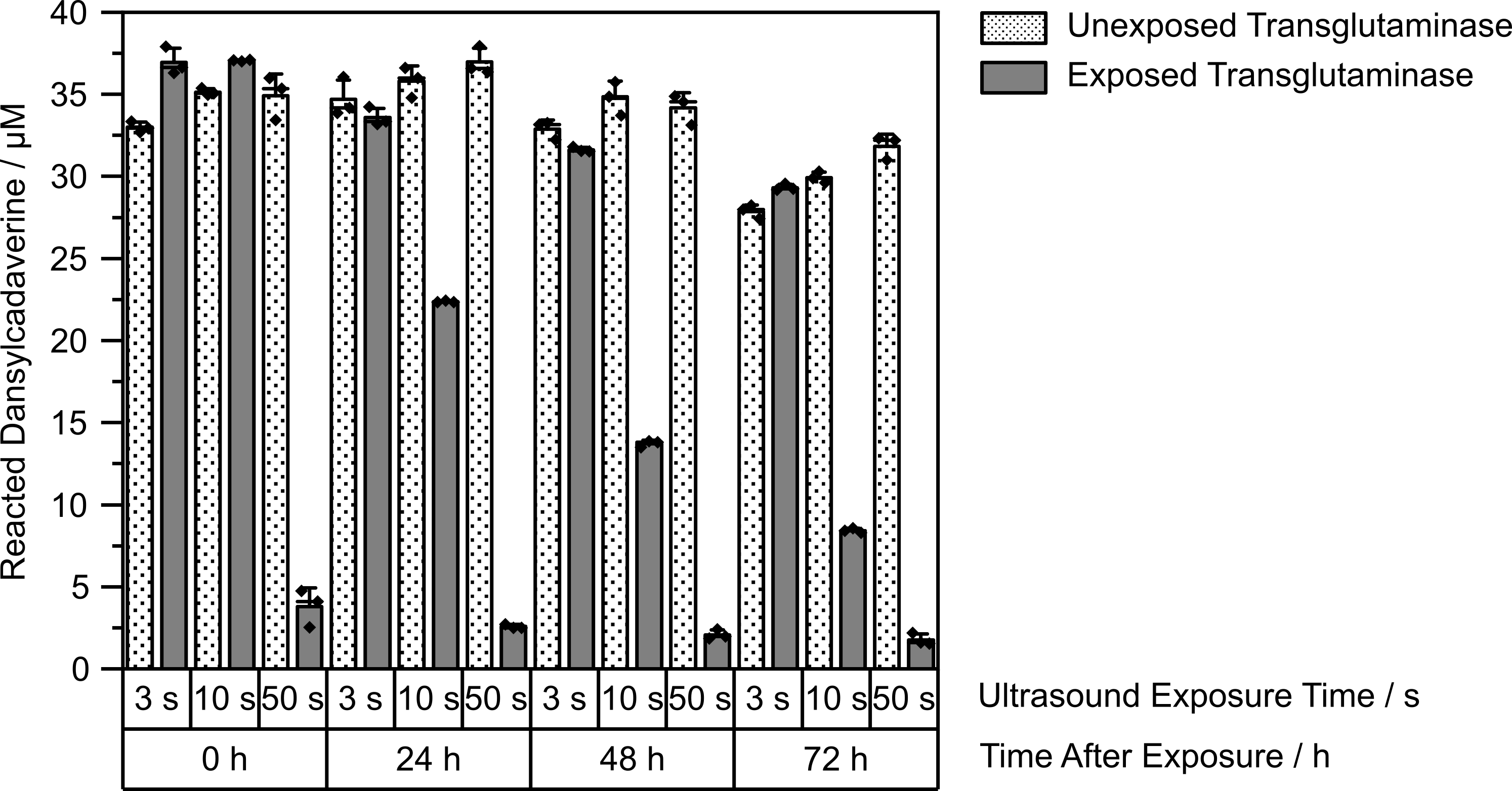


**Figure S11.** The effect of ultrasound exposure upon transglutaminase activity. A mixture containing transglutaminase and calcium-loaded liposomes was exposed to ultrasound for 3, 10 or 50 s. The substrate mixture (dimethylcasein and dansylcadaverine) was added at different time points post-exposure (0, 24, 48 and 72 h) and the fluorescence intensity at 505 nm was recorded after 24 h incubation with the substrate (dark gray bars). As a control, calcium-loaded liposomes were exposed to ultrasound for 3, 10 and 50 s and mixed with unexposed transglutaminase and the substrate mixture at the corresponding time points (light gray bars). This showed a decrease in enzyme activity for the 50 s exposure at each measured time point, and a decrease in enzyme activity for the 10 s exposure in the samples incubated for more than 24 h. The 3 s exposure showed retained activity, compared to the unexposed control, throughout. Data shown as mean ± S.D., n = 3.

**
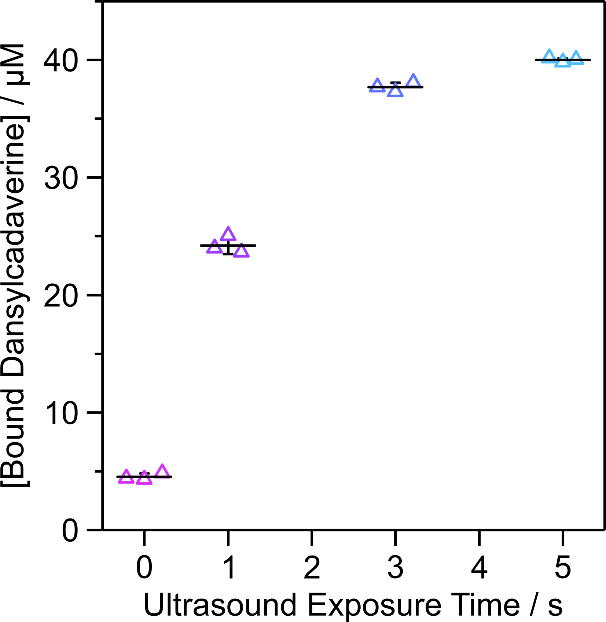
**

**Figure S12.** Ultrasound-triggered transglutaminase catalysis. 21 h endpoint measurements of the bound dansylcadaverine after transglutaminase, dansylcadaverine and calcium-loaded liposomes were exposed to ultrasound for 0, 1, 3 and 5 s. Data shown as mean and standard deviation of three technical replicates from the same batch of sonicated components.


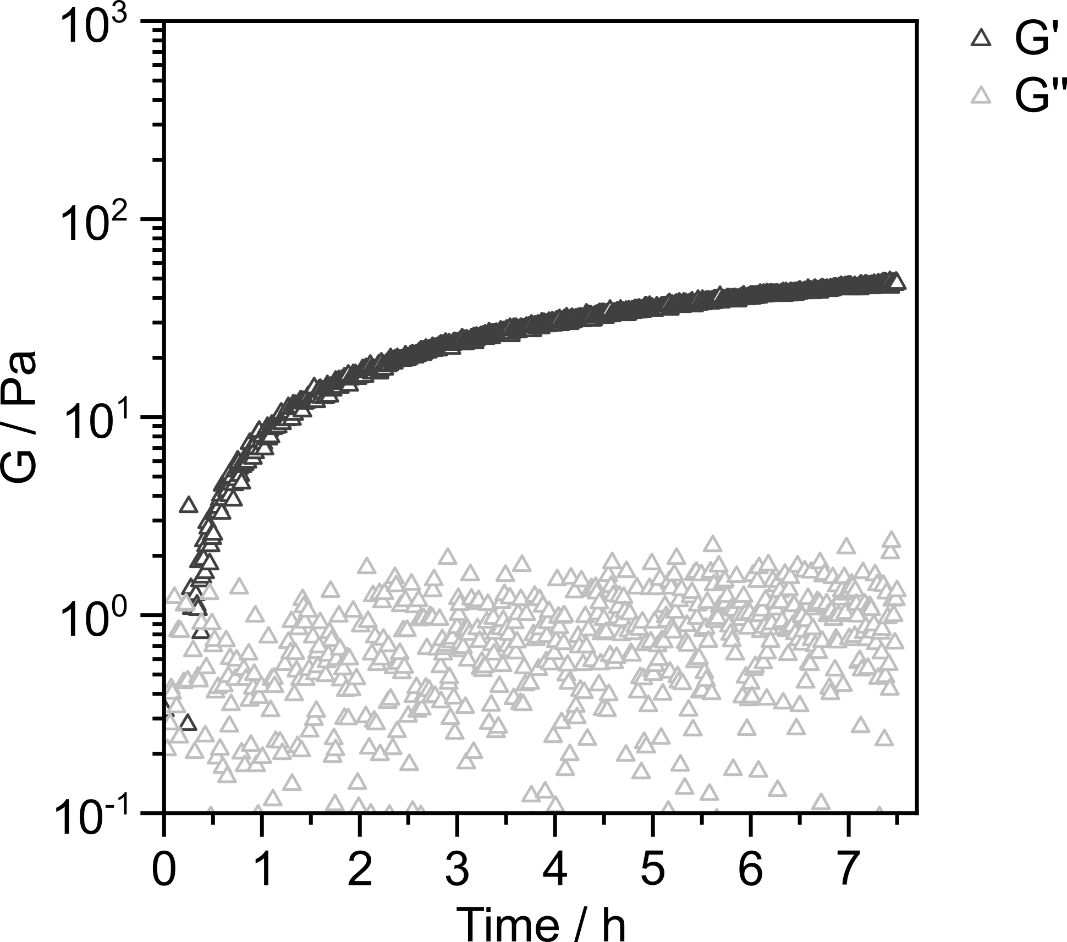


**Figure S13.** One-pot ultrasound-triggered fibrinogen hydrogelation. Ultrasound was applied for 10 s to a mixture of fibrinogen (final concentration of 22.4 mg mL^-1^), calcium-loaded liposomes and transglutaminase (final concentration of 5 µM). Time-sweep rheometry using 1% strain and 1 rad s^-1^ was performed at 25 °C. It should be noted that there was a lag period of approximately 10 minutes between the ultrasound stimulation and the rheological measurement.


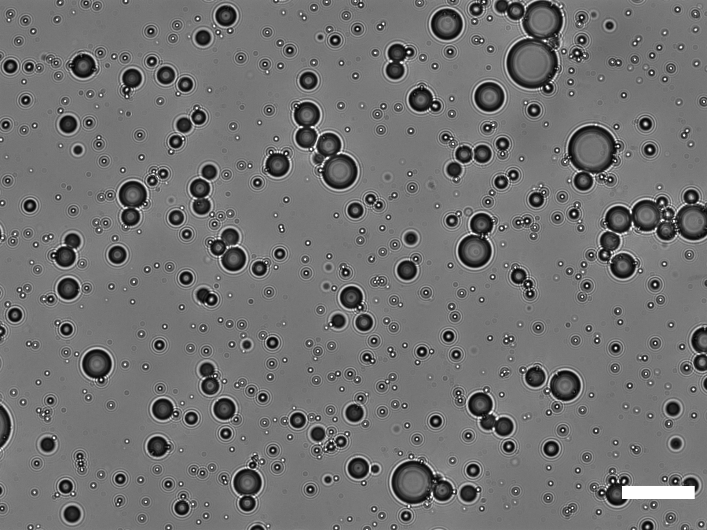


**Figure S14.** Bright field microscopy of DSPC / DSPE-PEG_2000_ / DSPE-PEG_2000_ biotin microbubbles. Scale bar: 20 µm.

**
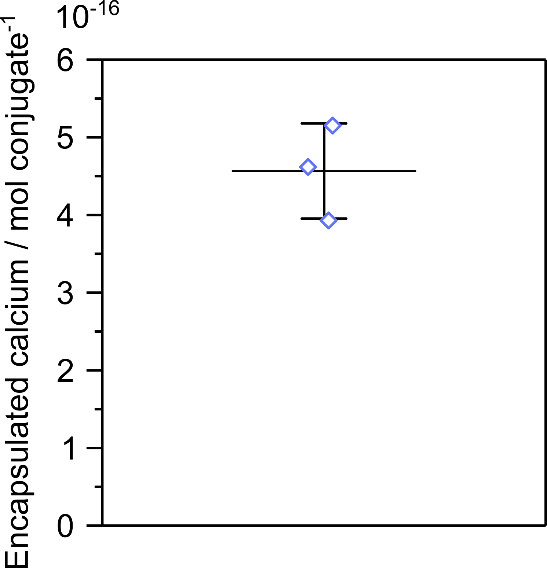
**

**Figure S15.** Microbubble-liposome conjugate calcium encapsulation. The total calcium was measured using an o-CPC assay and then normalized by the microbubble-liposome concentration. Data shown is the mean and standard deviation of three independent batches of conjugates.
